# The Importance of Understanding COVID-19: The Role of Knowledge in Promoting Adherence to Protective Behaviors

**DOI:** 10.3389/fpubh.2021.581497

**Published:** 2021-04-06

**Authors:** Lisa M. Soederberg Miller, Perry M. Gee, Rachael A. Katz

**Affiliations:** ^1^Department of Human Ecology, University of California, Davis, Davis, CA, United States; ^2^Intermountain Healthcare, Clinical Operations, Salt Lake City, UT, United States

**Keywords:** COVID-19, protective behavior, prior knowledge, essential workers, pessimistic illness expectations

## Abstract

**Background:** Past research suggests that knowledge supports- but strong illness expectations thwart- adoption of protective behaviors (e.g., handwashing). Strong illness expectations may place COVID-19 essential workers at risk. It is unclear, however, whether knowledge can moderate the negative effects of pessimistic illness expectations on protective behaviors. We test COVID-19 knowledge as a moderator of the effects of (1) pessimistic illness expectations and (2) essential worker status on adherence to protective behaviors.

**Methods:** Participants (*n* = 350) completed measures of knowledge, illness expectations, and protective behaviors. We used chi-square tests to examine associations between variables and logistic regressions to test the moderation models predicting adherence (low, high) while controlling for demographics.

**Results:** Knowledge, illness expectations, and adherence were significantly associated with each other (*p* < 0.05). Essential workers had stronger illness expectations and lower knowledge than did non-essential workers (*p* < 0.001). Logistic regressions showed a non-significant Worker Status × Knowledge interaction (*p* = 0.59) but a significant Knowledge × Illness Expectations interaction (*p* < 0.05) indicating that those with strong illness expectations and low knowledge were disproportionately at risk of failing to adhere to recommended behaviors.

**Conclusions:** Knowledge promotes protective behaviors by buffering the negative effects of pessimistic illness expectations. Essential workers are more likely to have low levels of knowledge with strong illness expectations, suggesting that educational policies may be warranted.

## Introduction

The COVID-19 pandemic is having devastating effects on human health and well-being and will likely continue to do so through its negative impact on the economy and poverty (1). The magnitude of the crisis can make it difficult to recognize the fact that individuals play an important role in slowing the spread of infection. Protective behaviors, sometimes called non-pharmaceutical interventions (NPIs), such as social distancing and handwashing, are critical to limiting the spread of infectious diseases (2–4). Essential workers, those who provide critical goods and services during the pandemic, often occupy low-wage positions in public transportation, food production, retail of food and health supplies, and healthcare. Protective of essential workers is particularly important given they are likely to have greater exposure to the virus and are at greater risk of financial strain if they do become infected (5–7).

The Common-Sense Model of Self-Regulation argues that the processes underlying individuals' conceptualization of an illness, referred to as an illness-related memory schema or mental model, include perceptions surrounding the threat posed by the illness and inform potential responses to the threat (8–11). The framework is typically applied to situations in which the patient has experienced a symptom of the illness but is also applicable to self-regulation of prevention-related behaviors during a pandemic. Specifically, illness-related memory schema are based on knowledge and beliefs about the illness and play an important role in the adoption of protective behaviors.

Past research on communicable diseases supports this notion by showing that knowledge and beliefs are important predictors of behaviors that impact the spread of the disease. For example, prior knowledge of a disease has been shown to increase handwashing, which in turn limited the spread of disease (12) and increase willingness to forgo public activities (11). In addition, misunderstandings (i.e., knowledge deficits) about influenza reduced adoption of protective behaviors (13–15). A recent study on COVID-19, on the other hand, reported no effects of knowledge on NPI, which as the authors noted, could be due to overall high knowledge scores (16) Another study, conducted when physical distancing but not mask-wearing was highly recommended (17, 18), found that higher levels of COVID-19 knowledge were associated with attending fewer large gatherings and not wearing a mask when leaving home (19).

In general, the research above suggests that knowledge supports effective health-related decision making. This is consistent with the expression “knowledge is power,” which has appeared in cognitive sciences for decades to illustrate the importance of knowledge in human and artificial intelligence (20). Theories, such as the Long-term Working Memory theory (21), propose that the advantages are due to knowledge structures that facilitate comprehension of- and memory for—information that is germane to the knowledge domain (22–24).

In contrast to the beneficial effects of knowledge on NPIs, research indicates that some types of illness-related beliefs can interfere with the adoption of protective behaviors. Specifically, a high level of certainty that one will become infected is associated with lower adherence to health-protective behaviors (25–28). Strong illness expectations may represent the belief that fate, rather than the individual, controls whether the individual contracts the illness, making protective behaviors relatively unimportant (25, 26). This is consistent with the notion that pessimistic, or *why bother*, beliefs increase avoidance behaviors (11, 29–31). On the other hand beliefs, such as perceived vulnerability, are positively associated with protective behaviors, which presumably help to reduce discomfort associated with feeling vulnerable (32, 33).

We are not aware of any studies on protective behaviors that have assessed both knowledge and beliefs as well as the relationship between knowledge and beliefs. However, a recent study that took place prior to an outbreak of COVID-19 in Australia included both knowledge and beliefs as predictors of NPI and vaccine intentions (34). Results showed that beliefs, including self-protection efficacy and perceived vulnerability, were positively associated with NPI but neither predicted vaccination intentions. Knowledge (symptoms, transmission, and general knowledge) was negatively associated with NPI but positively associated with vaccination intentions (34). Given the timing of the study, it could be that knowledgeable individuals understood that the threat had not yet reached a critical level so NPIs were not prioritized. Another challenge with interpreting the knowledge findings is many of the items were in areas that were rapidly evolving, making it difficult to know whether an individual knew more or less than what had been released to the public at that time (34). In another recent COVID-19 study, researchers showed that providing expert information about coronavirus infectiousness reduced fatalistic beliefs (35). Although NPIs were not assessed in that study, the findings suggest that providing knowledge can reduce maladaptive beliefs. Thus, despite theoretical work supporting the notion that mental models of one's illness, comprised of knowledge and beliefs, play an important role in health behaviors (8–11), there is little direct evidence regarding the extent to which knowledge mitigates the negative effects of illness expectations on the adoption of protective behaviors.

In the present study, we examined the extent to which COVID-19 knowledge and illness expectations predicted adherence to protective behaviors (handwashing, wearing a mask, avoiding crowded areas, 6-foot distance between individuals). We anticipated that knowledge would be positively associated with—but that illness expectations would be negatively associated with—protective-behavior adherence. The current pandemic differs from many past outbreaks in the US in that most individuals were ordered to stay-at-home at the time this study took place (18), while essential workers were not, providing an opportunity to compare knowledge and beliefs of essential and non-essential workers. We expected that essential workers may have stronger illness expectations than non-essential workers. It is unclear, however, whether knowledge would differ between essential and non-essential workers. Finally, the extent to which knowledge protects against disruptive beliefs, knowledge would be expected to moderate the relationship between beliefs and adherence to protective behaviors, and possibly, between essential-worker status and protective behaviors.

## Methods

### Participants

Participants (*n* = 350) from across the United States were recruited through Amazon Mechanical Turk (MTurk) during the 2nd week of April 2020. Stay-at-home mandates were in place for the majority of states at that time (six states had recommendations only, one state had neither a mandate nor recommendation) (18). MTurk is a crowdsourcing platform that is appearing with increasing frequency in behavioral and medical research (36, 37). This method allows individuals to participate from home at any time of day, which may have been particularly advantageous during the pandemic. The study was approved by the university's IRB.

### Measures

To assess knowledge related to COVID-19, we created 15 True/False questions from public-facing information provided on Johns Hopkins Medicine website on basic definitions and common myths about COVID-19. We developed this measure because no knowledge tests existed at the time of the study. We included items that assessed general information about the virus relative to other infectious diseases, virus transmission, and prevention (38). Items and correct answers are presented in Table 1. With one exception (*There is no vaccine to protect against the virus*), answers to the knowledge questions did not change between the time the study took place and the publication of this paper. A vaccine was developed roughly 7 months following the study. The total number of correct responses was used in the logistic regression analyses; however, for consistency with other predictors, a categorical variable (based on a median split) was used to examine unadjusted relationships. Pessimistic illness expectations were assessed using two items: *To what extent do you expect to become – infected with COVID-19/– very sick if infected* on a scale of 1 (Definitely will not) to 5 (Definitely will) (39). Because we were interested in pessimistic illness expectations, we considered high scores (ratings of 4 or 5) on either or both items to indicate strong illness expectations and low scores (ratings of 1–3, which included neutral expectations) on both items to indicate weak expectations.

**Table 1:** Knowledge items by response type percentage and correct responses shown in bold.

	**True**	**False**	**Not sure**
The virus is a severe form of the flu	43.4	**44.3**	12.3
Pets can spread the virus to humans	29.0	**48.4**	22.6
The virus spreads more quickly than most others including SARS	**78.9**	11.1	10.0
The virus is a mutated form of the common cold	27.3	**55.4**	17.3
Social distancing is key to reducing the spread of the virus	**83.6**	12.9	3.5
Individuals without symptoms can spread the virus	**79.5**	16.1	4.4
The virus can spread through insect bites	16.7	**69.8**	13.5
There is no vaccine to protect against the virus^*^	**83.0**	12.9	4.1
The primary, overarching goal of requiring people to shelter in place is to decrease the rate of transmitting the virus	**60.1**	39.9	NA
There are different kinds of coronaviruses, all of which can cause serious illness in humans.	70.1	**18.5**	11.4
The coronaviruses are named for their smooth surface as seen under a microscope.	24.0	**52.5**	23.5
Health officials do not believe COVID-19 was deliberately created or released by people.	**58.7**	20.2	21.1
The virus can cause severe respiratory problems impacting the nose, throat, and lungs.	**91.2**	5.9	2.9
The incubation period of COVID-19 is within 14 days of initial symptoms.	88.3	**6.2**	5.6
At this time, the number of people who have died from COVID-19 worldwide is far lower than the number of people who have recovered.	**66.6**	16.4	17.0

* *A vaccine had not yet been developed in April, 2020, when the survey was administered; NA, not applicable because this item had only True/False options*.

Protective behaviors were assessed by asking participants how frequently they followed 9 recommendations put forth by CDC (e.g., washing hands, social distancing) on a scale of 1 (Not Often) to 4 (Always) to reduce their risk of getting or spreading COVID-19 (2). *Adoption* to a protective behavior was considered *Yes* for scores of “often” or “always” with high adherence defined as the adoption of all nine behaviors. The items, shown in Table 2, created a reliable assessment of overall protective behaviors as reflected in a Cronbach's alpha coefficient of 0.80. To assess essential worker status, we asked individuals to indicate whether they were an essential worker, non-essential worker, or not working. Finally, we asked participants whether they had tested positive for COVID-19 as a covariate.

**Table 2:** Adherence to protective behaviors.

	**Count**	**%**
Washing hands and/or using sanitizers frequently	283	83.0
Staying at least 6 feet away from others	288	84.5
Avoiding large gatherings	299	87.7
Not going out to restaurants or bars	277	81.2
Wearing a face mask when outside the home	212	62.2
Not shaking hands or touching people	288	84.5
Wiping down surfaces with disinfectant	231	67.7
Staying at home (except for buying food, etc)	289	84.8
Limiting contact with others	290	85.0

### Analytic Strategy

Unadjusted associations among essential worker status (yes/no), knowledge (low, high), illness expectations (weak/strong pessimism), and protective behavior adherence (low, high) were examined using Chi-square and Spearman's rho. We conducted logistic regressions to test two moderation models on adherence after controlling for age, sex, race, ethnicity, education, and income. The first model tested illness expectations as a possible moderator of the effects of knowledge on prevention behaviors and the second model tested worker status as a possible moderator. In both models, knowledge scores (i.e., total number of questions correctly answered) were mean-centered prior to creating the interaction term.

## Results

Of those enrolled in the study (*n* = 350), 9 failed to pass the attention check and were excluded from analyses. As shown in Table 3, the final sample (*n* = 341) was 40.2% female, 78.6% Caucasian, and generally well-educated with 62.5% having 2 or more years of college. Close to one-third of the sample (36.7%) were essential workers; only six participants indicated they were not working and these individuals were included in the non-essential worker group. Essential workers were more likely to be Hispanic (*p* < 0.001), but did not differ in terms of age ($\chi_{1}^{2}$ < 1), sex ($\chi_{1}^{2}$ = 1.30, *p* = 0.28), race ($\chi_{1}^{2}$ = 5.01, *p* = 0.08), education level ($\chi_{1}^{2}$ = 1.62, *p* = 0.20), or income ($\chi_{1}^{2}$ < 1). The null finding for income is contrary to the suggestion that non-essential workers earn less than other workers (5), and could be due to the relatively well-educated individuals who tend to participate in research through online panels.

**Table 3:** Participant characteristics (*n* = 341).

**Variable**	**Description**	***N***	**%**
Age (years)	20–35	174	51.0
	35–73	167	49.0
Sex	Male	204	59.8
	Female	137	40.2
Education	<2 years of college	128	37.5
	≥2 years of college	213	62.5
Race	Caucasian	268	78.6
	Non-caucasian	73	21.4
Ethnicity	Hispanic	42	12.3
	Non-hispanic	299	87.7
Income	< $50,000	140	41.1
	≥$50,000	201	58.9
Essential worker	No	209	61.3
	Yes	132	38.7
Pessimistic illness expectations	Weak	206	60.4
	Strong	135	39.6
Adherence to protective behaviors	Low	216	63.3
	High	125	36.7
	**Range**	**Mean (SD)**	
Knowledge (0–15)	3–15	9.15 (2.44)	

Overall, adherence to protective behaviors was high as indicated by adherence rate of 80% across the nine NPI behaviors. Close to two-thirds of the sample (63%) reported adherence to eight or fewer behaviors. The distribution was highly skewed to the left (skewness = −1.45), leading us to dichotomize the distribution into partial adherence (low) and complete (high) adherence, which represented the top third of the distribution with adherence to all nine behaviors (40). As shown in Table 2, the behavior with the lowest adherence was wearing a face mask when outside the home (62%) and the behavior with the highest adherence was avoiding large gatherings (87%).

### Unadjusted Associations

Essential worker status was associated with lower knowledge and more pessimistic illness expectations (*p* < 0.001 for both). Higher knowledge was associated with less pessimistic illness expectations (*p* = 0.02). Adherence was associated with higher knowledge (*p* = 0.04), non-essential worker status (*p* < 0.01), and less pessimistic illness expectations (*p* < 0.01). Although not a key variable, it is interesting to note that only 14 participants (4.1%) indicated that they had tested positive for COVID-19; all of these individuals were essential workers and 13 (92.8%) were in the low-adherence group.

### Logistic Regressions

We tested the fit of two moderation models using logistic regressions. For both models, demographic variables (age, sex, race, ethnicity, education, and income) were added in block 1, main effects of key variables (essential worker status, illness expectations, and knowledge) and virus test results were added in block 2, and the interaction effect Knowledge x Illness Expectations (Model 1) or Knowledge x Worker Status (Model 2) was entered in block 3 (see Table 4).

**Table 4:** Tests of model effects predicting adherence to protective behaviors (significant effects shown in bold).

	**Variable (reference group)**	**B**	**S.E**.	**Sig**.	**Exp(B)**	**95% C.I. for EXP(B)**	
						**Lower**	**Upper**
Block 1	Age (20–35)	−0.45	0.24	0.06	0.64	0.40	1.03
	Sex (male)	0.36	0.24	0.14	1.43	0.90	2.29
	Education (<2 years college)	0.24	0.25	0.33	1.28	0.78	2.09
	Hispanic (non-hispanic)	−0.28	0.38	0.46	0.76	0.36	1.59
	Race (Caucasian)	0.09	0.29	0.75	1.10	0.63	1.92
	Income (< $50,000)	**0.59**	**0.25**	**0.02**	**1.81**	**1.10**	**2.97**
Block 2	Essential worker status (no)	**−0.55**	**0.27**	**0.04**	**0.57**	**0.34**	**0.97**
	Pessimistic illness expectations (weak)	−0.48	0.26	0.06	0.62	0.37	1.03
	Knowledge	0.06	0.05	0.24	1.06	0.96	1.17
	Tested positive for covid-19 (no)	−1.64	1.10	0.14	0.19	0.02	1.67
Model 1 - Block 3	Knowledge by illness expectations	**0.26**	**0.11**	**0.02**	**1.29**	**1.04**	**1.60**
Model 2 - Block 3	Knowledge by worker status	−0.58	0.11	0.59	0.94	0.76	1.16

*Total Nagelkerke R^2^ Model 1 = 0.14; Model 2 = 0.12 (R^2^ Block 1 = 0.05, R^2^ Block 2 = 0.12)*.

**Figure 1:**
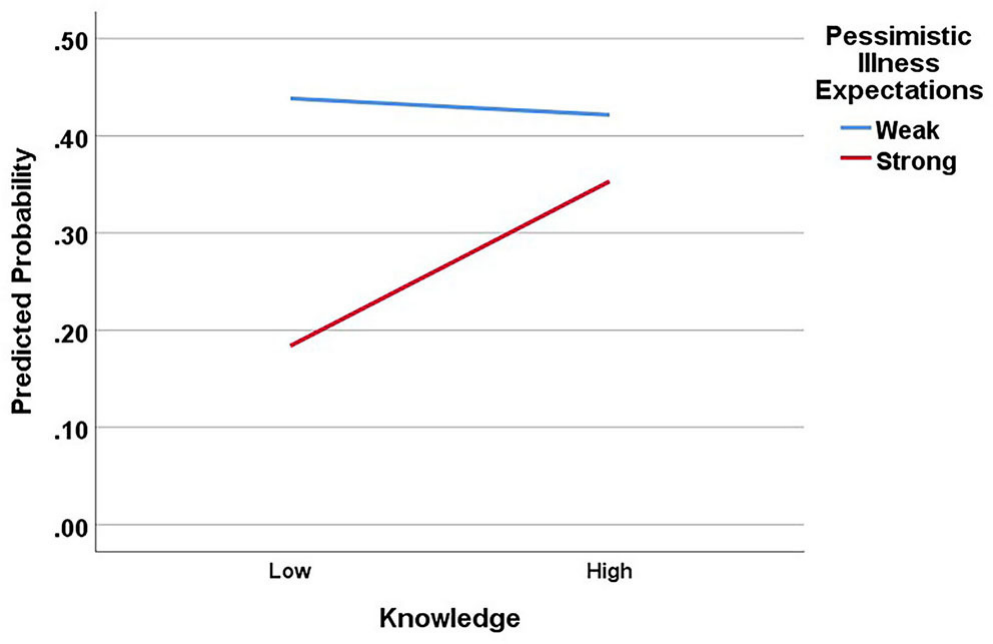
Moderating effects of knowledge on the relationship between pessimistic illness expectations and adherence to protective behaviors. Note, knowledge scores were entered as a continuous variable in regression analyses but are shown here as a dichotomous variable (median split) for illustration purposes.

Data from Block 2 reflect the effects of predictors after controlling for demographic variables. Results showed Essential Worker Status was negatively associated with adherence (OR 0.58, 95% CI 0.34–0.97, *p* = 0.04) but the effects of illness expectations (OR 0.62, 95% CI 0.37–1.03, *p* = 0.06) and knowledge (*p* = 0.24) were not significant. Because the zero-order associations were significant, the non-significant effects are likely due to variance shared with the variables entered in blocks 1 and 2. Block 3 differed for each model. In Model 1, the Knowledge × Illness Expectations interaction was significant (*p* = 0.02). Figure 1 shows the predicted values from the model indicating that the negative association between pessimistic illness expectations and adherence was evident for those with lower levels of knowledge only. The Essential Worker Status × Knowledge interaction was not significant (*p* = 0.59), indicating that knowledge moderates the effects of illness expectations, but not essential worker status *per se*.

## Discussion

Findings from the present study are consistent with past research on NPIs indicating that knowledge is positively—and pessimistic illness expectations are negatively—associated with protective behaviors (12–14, 25, 26). We add to the literature by showing that knowledge and illness expectations are negatively associated with each other and suggest that the two predictors have opposing effects on adherence to protective behaviors. We further specify the nature of the relationships by showing that knowledge moderates the effects of illness expectations on adherence such that the negative effects of high levels of illness expectations as mitigated by high levels of knowledge. We cannot determine from this cross-sectional study whether illness expectations lead to adherence failure or knowledge leads to adherence success. Nor can we determine how knowledge impacts the relationship between illness expectations and adherence. Although it seems plausible that understanding the virus tempers the certainty that one will become infected if some precautions are taken, additional research is needed to examine causal links.

The findings are consistent with the Common-Sense Model of Self-Regulation arguing that knowledge and beliefs play a critical role in illness representations (or schema), which in turn drive behavior (9). Illness expectations that are constructed from knowledge about the virus, how it is transmitted, and what limits transmission may protect against potentially harmful beliefs based on misunderstandings and mistrust of credible sources. For example, understanding that a rapid rate of transmission could overwhelm the healthcare system and in turn limit care for everyone, not just those with COVID-19, may prevent individuals from believing that personal choice should dictate adherence to protective behaviors (41). Thus, knowledge-based illness representations may serve as a comprehensive navigation tool for making effective health-related decisions during the pandemic (38).

An important question to consider in future research is how rapidly changing scientific knowledge of an infectious disease impacts the acquisition of laypersons' knowledge of effective NPIs. With many unknowns about the novel coronavirus, particularly at the start of the pandemic, scientific evidence and therefore NPI recommendations were in flux. For example, recommendations to use face covering, broadly defined, appeared at the end of March, 2020; whereas the more precise recommendation to use of multi-layer cloth masks appeared in November, 2020 (17). State and county mandates surrounding masks and other NPIs have also shifted over time, potentially affecting acceptance of NPIs among the public, and subsequently, COVID-19 growth rates (42). The flow of information between public health officials and the public is also influenced by social media, which includes information that extends beyond geo-political boundaries (43, 44).

Still, even under stay-at-home orders, individuals have many opportunities to be around others inside and outside the home (e.g., visit others, grocery store) requiring the use of protective behaviors. The abundance of misinformation that occurred during COVID-19 has made the question of protection against incorrect information more salient. Future research is needed to examine the extent to which science literacy could serve as a buffer against misinformation that threatens the public's health and well-being.

The rapid spread of COVID-19 in the spring of 2020 likely increased fear and confusion surrounding safety and may have decreased the opportunity to acquire factual information about the virus, for example, its incubation period and transmission process. Layered on top of this, the coronavirus has a relatively wide window of time, potentially 2 weeks, in which those who are infected with COVID-19 can transmit the virus without being aware that they are infectious (45). Thus, targeted strategies to increase individuals' understanding of COVID-19 may be a necessary component of an organization's safety plan as well as public health outreach more generally.

The data showing that (1) essential workers had strong illness expectations and low levels of knowledge and (2) both patterns predicted reduced adherence to protective behaviors suggest an additional layer of vulnerability. When essential workers—and those they serve—fail to adhere to protective behaviors, risk increases for all. It is unclear what should be done when essential workers or the public fail to adhere to orders requiring protective behaviors. However, an equally important question may be how do we promote learning about COVID-19 and other infectious diseases as a way to prevent adherence failures. Research is needed to examine the extent to which knowledge reduces the impact of maladaptive beliefs on NPIs as well as pharmaceutical interventions such as vaccinations, which are being avoided by a growing number of individuals (46, 47).

### Limitations

Limitations of this study include the use of crowdsourcing panel that is predominantly white and relatively well-educated and the majority of participants had some college education. Given that education would be expected to increase adherence, the findings may provide a more optimistic view of adherence than is warranted. However, the lack of ethnic and racial diversity limits the generalizability of the findings. Additionally, the study assessed knowledge, illness expectations, and protective behaviors at only one point in time and it could be that these factors change as the crisis evolves. Another limitation is that the study did not differentiate among types of essential workers, such as healthcare or food service, or consider official designations of essential worker categories at the time of data collection. It seems likely, for example, that healthcare workers who interacted with a volume of patients could have greater illness expectations or higher knowledge than other essential workers. Finally, it is important to recognize that the sample size of the study was small relative to epidemiological studies and was not representative of the population. The study was intended to provide an exploration of the dynamics between knowledge and beliefs within a context of a growing pandemic to consider how these factors could potentially impact NPIs. Replication with a larger, representative sample is needed to build on these findings, further specify mechanisms underlying adherence to protective behaviors, and inform the development of interventions that seek to empower individuals through increased knowledge and decrease pessimistic illness expectations.

## Conclusions

This study indicates that pessimistic illness expectations increase the risk of failing to adhere to protective behaviors but that knowledge protects against the negative effects of these expectations. The findings have implications for practice and policy, particularly related to essential workers and their environment. Additional work is needed to identify optimal approaches to increasing individuals' knowledge to the point where it reduces or eliminates maladaptive beliefs. By helping to specify the predictors associated with protective behaviors during the pandemic, this line of inquiry may help to fill important gaps in our understanding of how to help slow the transmission of COVID-19 from individual to individual.

## Data Availability Statement

The raw data supporting the conclusions of this article will be made available by the authors, without undue reservation.

## Ethics Statement

The studies involving human participants were reviewed and approved by IRB University of California, Davis. The patients/participants provided their written informed consent to participate in this study.

## Author Contributions

LM conceptualized the study and collected and analyzed the data. All authors contributed to drafting and revising the manuscript.

## Conflict of Interest

PG and RK were employed by the company Intermountain Healthcare. The remaining author declares that the research was conducted in the absence of any commercial or financial relationships that could be construed as a potential conflict of interest.
